# Temporal Quasi-Phase Matching Assists Robust Acoustic Adiabatic Passage

**DOI:** 10.34133/research.0362

**Published:** 2024-05-09

**Authors:** Klaas Bergmann

**Affiliations:** Retired from Department of Physics, Rheinland-Pfälzische Technische Universität (RPTU), Kaiserslautern, Germany.

## Abstract

Recent work demonstrated stimulated Raman adiabatic passage-type transfer of energy along 3 acoustic cavities. After brief comments on the stimulated Raman adiabatic passage method, remarks on the scientific and technological relevance of this work are presented, followed by noting other recent important applications of the process.

Recently, Chen and colleagues [1] from the Nanjing University, jointly with Xue-Feng Zhu’s group at the Huazhong University of Science and Technology, realized robust acoustic energy transfer between 3 acoustic cavities with different resonance frequencies by generalizing the quasi-phase matching to the time domain. The robustness of the process is attributed to the implementation of the stimulated Raman adiabatic passage method (STIRAP), a well-established technique with broad applications in both the quantum and classical regimes.

First, a brief discussion “STIRAP-in-a-nutshell” is offered, starting with reference to a system of quantum states (QSs) (see Fig. 1A). The task is to transfer the entire population of QS1 to an initially unpopulated QS3. The transfer should be efficient (nearly 100%) and selective, i.e., no other QSs should receive population during the transfer process. Direct single-photon radiative coupling between QS1 and QS3 is often not possible or not robust. Therefore, 2-photon coupling via an intermediate QS2 is implemented. To achieve the required strong coupling, this is best done with on-resonance tuning of the laser frequencies. However, whenever population reaches QS2, a large fraction of the population is lost by spontaneous emission to other QSs, summarized as QS4. The surprising message of the original STIRAP work [2] is that efficient and selective transfer is possible when initially only the unpopulated QS2 and QS3 are radiatively coupled by laser S. The radiative coupling of QS1 and QS2 by laser P starts rising not before the coupling induced by laser S has reached its maximal strength and begins to decrease (see Fig. 1B).

**Fig. 1.**
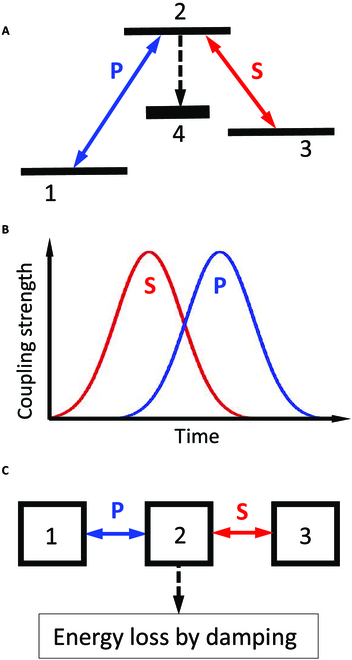
(A) Radiative coupling between QSs. (B) Time evolution of the coupling strengths. (C) Coupling between 3 acoustic cavities with different resonance frequencies.

The physics behind this “STIRAP-type” (counterintuitive) sequence of interactions is well understood [3]. The transition amplitudes, driven by the 2 lasers, interfere destructively so that no population is established in QS2. One requirement is that the process evolves adiabatically, i.e., it is not too fast [3]. Given the adiabatic evolution, the success of the transfer process is not sensitive to small variation in laser intensity or the time delay between the pulses of lasers S and P, meaning that the process is robust. It is, in particular, the latter remarkable feature that allowed STIRAP to become widely used in a multidisciplinary context.

The essence of the physics behind STIRAP is interference of amplitudes. Therefore, it is not a generic quantum process but applicable whenever waves are involved. To the best of our knowledge, this was first noted in theoretical work on the coupling of light transported through 3 closely spaced optical fibers [4] and experimentally shown in [5]. Equally astonishing, it was found that the physics also works for acoustic waveguide tubes with spatially suitably varying coupling between them [6]. Notably, all these waveguide implementations require identical elements, where the phase matching is naturally satisfied.

Now, Chen et al. [1] extend the STIRAP to arbitrarily detuned acoustic cavities (see Fig. 1C) in the time domain by demonstrating temporal quasi-phase matching. The acoustic resonance frequencies take the role of energy levels. The equivalence to the transfer of population between QS1 and QS3 is the transfer of energy of a resonant acoustic vibration in cavity 1 to cavity 3 via cavity 2, without appreciable excitation of a resonance in the latter. Therefore, loss of energy through acoustic damping in cavity 2 (the equivalent of spontaneous emission in the quantum system) is very small. Here, the role of lasers in the quantum system is taken by electronic coupling between the cavities. The amplitude of the acoustic wave in one cavity drives via a membrane an electric field through which, in turn, energy is deposited into the neighboring cavity. The difference of the resonance frequencies between the cavities is compensated by the temporal quasi-phase matching, i.e., by periodically switching the phase of the electric field, which drives the membrane depositing energy, by π. Like in the quantum system, the transfer is efficient when the coupling between the cavities 1 and 2 starts only after the coupling strength between the initially nonexcited cavities 2 and 3 has reached its maximum and begins to decrease. In the case of the quantum system, the electric field that drives the transfer varies typically at a femtosecond time scale. In the acoustic case, the relevant time scale is of the order of a tenth of a millisecond.

The work reported in [1] not only shows the applicability of the STIRAP method in the context of acoustic cavities but also demonstrates that the specific features of STIRAP, namely, relative simplicity of implementation and robustness against small variation of the system parameters, may have very important practical application. Inherent in the device presented in [1] is an “acoustic diode”, i.e., efficient energy transfer is realized along one path, while the transfer via the reverse path is blocked. It appears quite possible, even if challenging, to significantly miniaturize the components and thus pave the way for new technological applications.

The work with acoustic cavities is yet another important addition to the long list of innovative STIRAP applications in a large range of fields, such as the formation of ultracold molecules (which started the new field of ultracold chemistry [7]). The process played also a significant role in lowering the measured upper limit for the electric dipole moment of the electron (relevant for the field of high-energy physics), the coherent manipulation of superconducting circuits (relevant for elements in solid-state based quantum information systems), and transfer of population between QSs of nuclei (showing the perspective for new types of energy storage systems) to give just a few examples. References to those works can be found in [8]. Further examples are STIRAP-type transfer between nanomechanical oscillators [9], transfer within doped solids [10] with applications to quantum information [11], and even transfer of particles along a chain of 3 spatially separated traps, predicted some 20 years ago [12,13] and recently experimentally realized [14].

The evolution of applications of the STIRAP process is an example for how surprising developments, triggered by results from basic research, can be. The original paper on STIRAP [2] was published in a physical chemistry journal because the motivation for that work was the plan to experimentally study the effect of vibrational excitation of some small molecules on reactions relevant to the chemistry in the atmosphere. Surprisingly, significant progress along those lines has not yet been achieved. However, the creativity of scientists like the ones of [1], contributed to establishing the STIRAP concept as a universal tool.
